# Extracellular Redox Regulation of Intracellular Reactive Oxygen Generation, Mitochondrial Function and Lipid Turnover in Cultured Human Adipocytes

**DOI:** 10.1371/journal.pone.0164011

**Published:** 2016-10-14

**Authors:** Albert R. Jones IV, Tova Meshulam, Marcus F. Oliveira, Nathan Burritt, Barbara E. Corkey

**Affiliations:** 1 Obesity Research Center, Department of Medicine, Boston University School of Medicine, Boston, Massachusetts, United States of America; 2 Laboratório de Bioquímica de Resposta ao Estresse, Instituto de Bioquímica Médica Leopoldo de Meis, Universidade Federal do Rio de Janeiro, Cidade Universitária, Rio de Janeiro, Brazil; Universidad Pablo de Olavide, SPAIN

## Abstract

**Background:**

Many tissues play an important role in metabolic homeostasis and the development of diabetes and obesity. We hypothesized that the circulating redox metabolome is a master metabolic regulatory system that impacts all organs and modulates reactive oxygen species (ROS) production, lipid peroxidation, energy production and changes in lipid turnover in many cells including adipocytes.

**Methods:**

Differentiated human preadipocytes were exposed to the redox couples, lactate (L) and pyruvate (P), β–hydroxybutyrate (βOHB) and acetoacetate (Acoc), and the thiol-disulfides cysteine/ cystine (Cys/CySS) and GSH/GSSG for 1.5–4 hours. ROS measurements were done with CM-H_2_DCFDA. Lipid peroxidation (LPO) was assessed by a modification of the thiobarbituric acid method. Lipolysis was measured as glycerol release. Lipid synthesis was measured as ^14^C-glucose incorporated into lipid. Respiration was assessed using the SeaHorse XF24 analyzer and the proton leak was determined from the difference in respiration with oligomycin and antimycin A.

**Results:**

Metabolites with increasing oxidation potentials (GSSG, CySS, Acoc) increased adipocyte ROS. In contrast, P caused a decrease in ROS compared with L. Acoc also induced a significant increase in both LPO and lipid synthesis. L and Acoc increased lipolysis. βOHB increased respiration, mainly due to an increased proton leak. GSSG, when present throughout 14 days of differentiation significantly increased fat accumulation, but not when added later.

**Conclusions:**

We demonstrated that in human adipocytes changes in the external redox state impacted ROS production, LPO, energy efficiency, lipid handling, and differentiation. A more oxidized state generally led to increased ROS, LPO and lipid turnover and more reduction led to increased respiration and a proton leak. However, not all of the redox couples were the same suggesting compartmentalization. These data are consistent with the concept of the circulating redox metabolome as a master metabolic regulatory system.

## Introduction

It is well established that oxidation-reduction (redox) reactions exist in all cells and that they play a major role in human health and disease. Redox reactions are important in energy metabolism, reactive oxygen species (ROS) generation, cell cycle regulation, cell growth, apoptosis, gene expression and aging [1–5].

ROS are derived from molecular oxygen and are produced in multiple cell compartments including mitochondria. When produced in large quantities, ROS have the potential to cause a number of deleterious events: but in small quantities, play a role in signaling. Both intracellular and extracellular thiol redox cycles play central roles in maintaining ROS/redox balance [6–8]

Normal cells must maintain a specific internal and external redox homeostasis, which is communicated among cells by blood metabolites [9]. The circulating redox metabolites are produced by cells and may be sensed by all the tissues of the body. These couples include: the thiol (SH/SS) reflected in the cysteine/cystine (Cys/CySS), the reduced and oxidized gluthathione (GSH/GSSG), and the pyridine nucleotides NADPH/NADP and NADH/ NAD. Pyridine nucleotides are equilibrated with lactate (L) and pyruvate (P) in the cytosol, and acetoacetate (A) and ß-hydroxybutyrate (βOHB) in the mitochondria (see review by Corkey and Shirihai [9]). The enzymes lactate dehydrogenase and ßOHB dehydrogenase are highly expressed in the cytosol and mitochondria, respectively. When the redox couples are out of the physiological range, redox metabolism is shifted and adverse reactions can occur [10,11].

In addition to protecting against damage caused by oxidative stress, thiol systems play an important role in cell signaling. A vast body of data has accumulated linking cellular redox homeostasis with aging that is associated with an oxidative shift in the thiol redox state of the intracellular glutathione pool and of the plasma cyst(e)ines [2,5,12]. In human plasma the GSH/GSSG and the Cys/CySS redox states are markers for oxidative stress during aging and age related diseases such as diabetic retinopathy, cardiovascular disease, chemotherapy, and cigarette smoking [11,13,14]. In normal human plasma, the redox potential of Cys/CySS is much higher (-80mV) than GSH and GSSG (-140mV). While a linear age dependent increase in the plasma Cys/CySS of 1.4 mV/year is recorded, the redox potential of GSH/GSSG does not change in the first 45 years but then becomes more oxidized by 0.7mV/year [10].

Findings with various cell types document functional responses to redox alterations. Human epithelial colorectal adenocarcinoma cells (Caco-2) were cultured at Cys/CySS redox states ranging from 0 mV to -150 mV and results showed that cell proliferation at the reduced redox potential was 2 fold higher than in the oxidized state. This stimulation occurred without an effect on the cellular GSH/GSSG ratio [13]. Similar results were obtained with endothelial cells: a more oxidized Cys/CySS ratio stimulated H_2_O_2_ but not NO production, activated NF-kB, increased expression of adhesion molecules and stimulated monocyte binding to endothelial cells [15].

Increasing evidence supports the hypothesis that many tissues play an important role in metabolic homeostasis and the development of diabetes and obesity. We hypothesize that the redox metabolome is a master metabolic regulatory system that also impacts adipocytes to modulate reactive oxygen species (ROS) production, lipid peroxidation, energy production and changes in lipid turnover as we have previously shown in liver [16].

In this study we used human adipocytes and acutely modulated extracellular redox state using L and P, βOHB and Acoc and the thiol-disulfide pairs Cys/CySS and GSH/GSSG, in different ratios over a physiological range. We monitored functional responses and determined whether the circulating redox state could impact human adipocyte metabolism. We found that changes in the external redox state regulated intracellular ROS production, LPO, energy efficiency, lipid handling, and adipocyte differentiation. The more oxidized state caused increased ROS, LPO and lipid turnover and a more reduced state lead to increased respiration and a mitochondrial proton leak.

## Material and Methods

Chemicals and reagents were purchased from Sigma-Aldrich (St. Louis, MO) unless otherwise noted. Clinical supplies and cell culture supplies were purchased from Thermo Fisher Scientific (Waltham, MA), Becton Dickenson (Hunt Valley, MD), or Invitrogen Life Technologies (Carlsbad, CA) unless otherwise noted.

The only commercial available form of acetoacetate was lithium acetoacetate, therefore lithium chloride control was used in this studies and found not to have an affect on any assay.

A 50:50 mixture of D-and L- β-hydroxybutyrate was used. Since only the D-enantiomer is convertible to Acoc by βOHB dehydrogenase, the concentration of βOHB was doubled.

### Human adipocytes

Human preadipocytes were provided by the Adipocyte Core of the Boston Nutrition Obesity Research Center (DK46200). Because cells were not linked with any identifying information, these experiments were not subject to review by the Institutional Review Board for human studies. The preadipocytes from five subjects were pooled and seeded in 24-well plates. Two days after reaching confluence they were differentiated as previously described [17]. Experiments were performed with cells between days 10–14 after differentiation unless otherwise noted.

### ROS measurements

ROS measurement are from the protocol described by Krawczyk et al. [18]. Briefly, cells were plated in 24-well plates at 2 × 10^4^ cells per well then grown and differentiated into mature adipocytes. On the day of the experiment, media were changed to Krebs Ringer Buffer (KRB) (130 mM NaCl, 4.7 mM KCl, 2.5 mM MgSO_4_, 3.3 mM CaCl_2_, 24.5 mM NaHCO_3_, 1 mM KH_2_PO_4_, 5 mM glucose incubated for 10–15 min with 5% CO_2_, 95% O_2_) containing 0.1% bovine serum albumin (BSA). Cells were incubated for 30 min at 37°C with 5% CO_2_ and 95% O_2_. ROS-sensitive dye, 2',7'-dichlorodihydrofluorescein diacetate (H2-DCF-DA; Invitrogen Molecular Probes, Eugene, OR), was added for 30 min at a final concentration of 5 μM. Cells were subsequently washed and test compounds were added in Hank’s Balanced Salt solution (HBSS). Fluorescent readings were acquired on a TECAN M1000 plate reader (Männedorf, Switzerland) every 2 min for 1.5 hours at 37°C with an excitation wavelength of 485 nm and an emission wavelength of 538 nm. Experiments were run for 1.5 hours based on previous work indicating that ROS reached a steady-state at this time [18]. Data were expressed as the relative change of the final value minus the initial baseline reading.

### Lipolysis

Lipolysis was measured as glycerol release as described previously [18,19]. Briefly, differentiated adipocytes were incubated in KRB with 0.5 mM oleate and 150 μM BSA with or without the test solutions for 4 hours. Forskolin (5 μM) was used as a positive control. Aliquots were removed and the glycerol content was measured using an NADH-linked assay as previously described [19]. Experiments were run for 4 hours due to the low sensitivity of the glycerol assay we used (as shown in our comparison of 1 and 4 hour incubation times in S1 Fig).

### Oxygen Consumption

Preadipocytes (1.5 X 10^4^/well) were seeded into each well of a V7 cell culture plate (Seahorse Bioscience, Billerica, MA), followed by cell attachment for 5 hours and thereafter addition of 200 μl medium (all media were as described [20]. Two days after reaching confluence they were differentiated as previously described [20]. Experiments were performed with cells between days 10–14 after differentiation. Before the O_2_ consumption measurements, maintenance media were replaced with a fresh media followed by incubation for 60 min at 37°C (no CO_2_) before loading into the XF24 extracellular analyzer (Seahorse Bioscience). Injection ports (A) were loaded with culture media, βOHB, or Acoc at final concentrations of 20 mM. (The only commercially available form of acetoacetate was a lithium salt therefore LiCl at 20 mM was used as control). A 10 μM solution of oligomycin A was loaded into injection ports B and used to inhibit ATP synthase by blocking the F0 subunit. In injection ports C, a 2.5 μM solution of FCCP to uncouple electron transport from ATP synthesis (maximum respiration). Finally, in injection ports D, a 10 μM solution of antimycin A was used to inhibit flux of reducing equivalents through the electron transport chain (minimum respiration). (All are final concentrations). Concentrations of inhibitors were selected by titration that produced optimal inhibition (data not shown).

### Lipid Peroxidation (LPO)

LPO was assessed in adipocytes using a modified TBARs assay based on a protocol by Ha *et al*. [21]. Briefly, cells were grown and maintained as for ROS measurements. Test compounds were added for 90 min (to match the time cells were treated in the ROS production assay). Cells were first washed with HBSS and then 250 μL of a solution containing 0.4% 2-thiobarbituric acid and 10% acetic acid was added. NaOH was added in a final concentration of 0.0625 N. Standard curves were produced by serial diluting 1,1,3,3-tetraethoxy-propane and treating standards the same way as the samples. The cell solutions and standard curve were incubated at 90°C for 60 min, then cooled and centrifuged at 15,000 *g* for 5 min. The supernatant was isolated and fluorescence was measured on the TECAN M1000 plate reader (Männedorf, Switzerland) with an emission wavelength of 553 nm and an excitation wavelength of 515 nm.

### Lipogenesis

Cells were pre-incubated for 30 min with KRB [18] and then switched to KRB containing 5 mM glucose. Cells were incubated for 4 hours in 5mM glucose with 13.5 μM uniformly labeled ^14^C-glucose. After the incubation period, cells were washed with PBS and lipids were removed using the Bligh-Dyer (chloroform-methanol) extraction [22]. Ten nM insulin and 10 μM forskolin were used as positive and negative controls. Samples were taken from the organic phase, suspended in scintillation fluid (Ecoscint, National Diagnostics) and radioactivity was measured using a liquid scintillation analyzer (TRI-CARB 2900TR Packard). Four hours incubation was selected to match lipolysis assay.

### Fatty Acid Oxidation measured by CO_2_ release

The assay was performed as previously described [23]. Briefly cells were pre-incubated for 30 min in KRB before incubated for 4 hours in KRB containing 5 mM Glucose and 25 μM of [1- ^14^C]-oleate (Concentration 50μCi/μmole, Perkin Elmer) in an airtight chamber. After the 4 hour incubation, the supernatant was collected and placed in a scintillation vial. A 1.5 cm filter paper (Whatman) was suspended above each vial with 15 μl of β-phenylethylamine and sealed to trap the CO_2_. CO_2_ was released following acidification of the media via the addition of 500 μl/vial of 6M H_2_SO_4_. The vial remained sealed for an additional hour in order to trap the ^14^CO_2_ produced during the incubation period onto the filters. Filter papers were collected and suspended in scintillation fluid (Ecoscint, National Diagnostics) and radioactivity was measured in a liquid scintillation analyzer (TRI-CARB 2900TR Packard).

### FA partitioning (esterification and released FFA)

The adipocytes were washed with PBS containing 12.5% Perchloric acid. The precipitate was centrifuged and resuspended in 200 μl of PBS, the cell suspension was extracted with chlorophorm methanol. Samples were taken from the organic and aqueous phases, suspended in scintillation fluid (Ecoscint, National Diagnostics) and radioactivity was measured in a liquid scintillation analyzer (TRI-CARB 2900TR Packard).

### Effect of GSH and GSSG on adipogenesis

Human preadipocytes in 24 well plates (2X10^4^ cells/well) were differentiated as detailed previously [17]. The media were supplemented with either 110 μM GSH or 55 μM GSSG The induction media were added on day 0 and the GSH/GSSG cocktail added either on day 0, 5, 9 or 12 of differentiation. All cells were differentiated for a total of 14 days and fat accumulation was assessed on day 14 by Oil Red O staining [24]. Briefly, on day 14 cells were washed with PBS, fixed with 3.7% formalin for 15 min and incubated with 0.3% dye in 100% isopropanol for 30–45 minutes. Cells were washed 5 times with sterile water and the dye was extracted with 4% NP-40 (in isopropanol) for 15 min then read at 520 nm in a TECAN M1000 plate reader (Männedorf, Switzerland)

### Statistical Analyses

Data are represented as mean ± SEM. P-values were calculated using ANOVA analysis with post-hoc Dunnetts test.

## Results

### A more oxidized extracellular redox state increased ROS production

**Fig 1:**
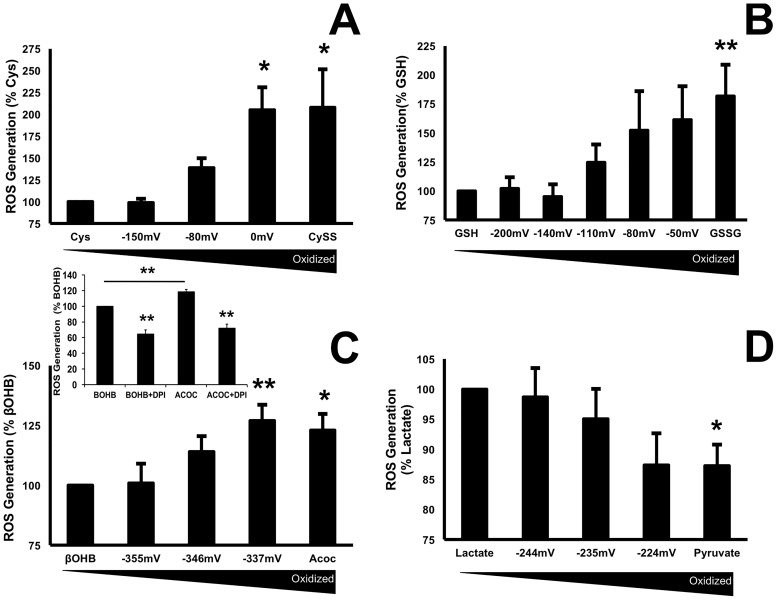
Extracellular oxidized ratios of redox couples increased ROS production. Human differentiated adipocytes were incubated with the hydrogen peroxide-sensitive dye 2',7'-dichlorodihydrofluorescein diacetate at a concentration of 5 μM. Cells were subsequently washed and the redox couples were added in Hank’s Balanced Salt solution (HBSS). Cysteine/cystine, and GSH/GSSG and their ratios are expressed as the steady-state redox potential calculated from the Nernst equation. A) 200 μM cysteine (cysteine + cystine), B) 110 μM glutathione (reduced + oxidized glutathione), C) 20 mM total active ketone with various ratios of ßOHB / Acoc (-355mV = 2:1 d-βOHB: Acoc; - 346mV = 1:1 d-βOHB: Acoc; -337 mV = 1:2 d-βOHB: Acoc as previously reported [16], C insert) 10 μM of the flavoprotein inhibitor, diphenyliodonium (DPI), significantly reduced levels of ROS generated by βOHB (p = 0.0002) and Acoc (p = 0.0008), D) 20 mM of L plus P with various ratios of L/P (-244mV = 20:1; -235mV = 10:1; -224mV = 5:1). Fluorescent readings were acquired on a TECAN M1000 plate reader every 2 min for 1.5 hours at 37°C with an excitation wavelength of 485 nm and an emission wavelength of 538 nm. Data are expressed as % change over the reduced species. Data were pooled from 3–4 independent experiments done in triplicate. Results are expressed as means ± SEM, ANOVA analysis with a post-hoc Dunnetts test was used for statistics. Values were Cys/Cyss p = 0.04 and 0.03, GSH/GSSG p = 0.01, ßOHB / Acoc p = 0.02 and 0.03 and for L/P p = 0.016 respectively.

We exposed differentiated human adipocytes to a physiological range of redox potentials for 1.5 hours using extracellular Cys/CySS, GSH/GSSG, βOHB/Acoc and L/P and studied the influence of these couples on ROS production using DCF as an indicator. Data showed that the extracellular addition of the most oxidized couples caused an increase of ROS that ranged from 123–202%, as illustrated for Cys/CySS (Fig 1A), GSH/GSSG (Fig 1B) and ketone bodies: βOHB/Acoc (Fig 1C). Addition of 10 μM of the flavoprotein inhibitor, diphenyliodonium (DPI), significantly reduced levels of ROS generated by βOHB (p = 0.0002) and Acoc (p = 0.0008) (Fig 1C insert). In contrast, the more oxidized metabolite pyruvate caused a 12% (p = 0.016) decrease in ROS compared with lactate (Fig 1D).

### The external redox state affected lipid peroxidation

**Fig 2:**
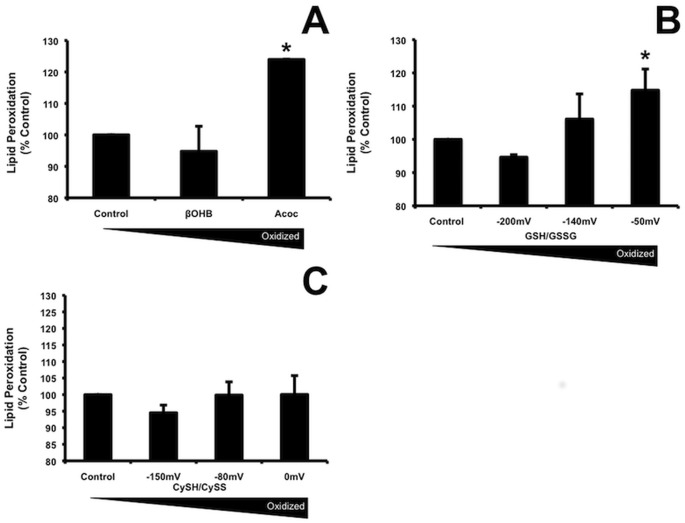
Extracellular oxidation by Acoc and GSH/GSSG but not Cys/CySS increased lipid peroxidation. LPO was assessed in adipocytes using modified TBARs. Cells were grown and maintained as for ROS measurements. Test compounds were added as in Fig 1 legend, for 90 min (to match the time cells were treated in the ROS production assay). LPO was measured as described in the materials and methods, A) Total concentration of active ßOHB plus Acoc was 20mM, B) GSH plus GSSG 110 μM glutathione (reduced + oxidized glutathione), C) 200 μM cysteine (cysteine + cystine). Data are pooled from 2 separate experiments done in triplicate. Results are expressed as % of reduced form. P = 0.02 comparing Acoc to ßOHB (mean ± SD: 122 ± 1.7%) and p = 0.046 comparing oxidized to reduced glutathione (mean ± SD: 115 ± 6.4%). Fifty μM tert-butyl hydroperoxide (tBH), an inducer of hydrogen peroxide production added as a positive control, increased lipid peroxidation (mean ± SD: 115.7 ± 8.3%) p = 0.03 n = 3 experiments. P-values calculated using ANOVA analysis with a post-hoc Dunnetts.

Reactive ROS intermediates can oxidize the polyunsaturated fatty acids present in cellular membranes leading to lipid peroxidation and a detrimental effect on membrane structure and fluidity. We therefore examined the effects of external redox state on lipid peroxidation. The degree of lipid peroxidation was estimated by monitoring the formation of thiobarbituric acid reactive substances (TBARS). Results in Fig 2A and 2B showed that, similar to ROS production, addition of the more oxidized species of Acoc and GSSG (-50 mV) increased LPO significantly (p = 0.02 and p = 0.046,), compared with their reduced species. Data are shown as mean ± standard deviation due to data being pooled from two experments. ANOVA analysis with a post-hoc Dunnetts test was used for statistics. The lower levels of lipid peroxidation induced by the reduced forms GSH and βOHB are consistent with their antioxidant activity. In contrast, the addition of more oxidized cystine (-0 mV) did not cause significant lipid peroxidation suggesting either distribution into different cellular compartments or different targets.

### Redox couples altered lipolysis

**Fig 3:**
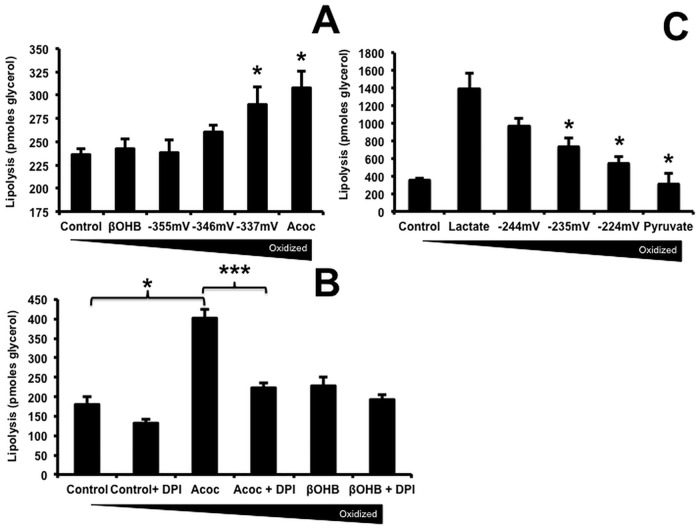
Redox couples altered lipolysis. Lipolysis was measured as glycerol release [18]. Adipocytes day 14 after differentiation were incubated in KRB with 0.5 mM oleate complexed to 150 μM BSA with or without the test solutions, as in Fig 1 legend, for 4 hours. Forskolin (5 μM) was used as a positive control (not shown). Aliquots were removed and the glycerol content was measured using an NADH-linked assay. DPI was added as an inhibitor of flavin oxidases at 10 μM (B). Data were pooled from 3 independent experiments done in triplicate. Results are expressed as means ± SD. A) Difference between ßOHB and Acoc was significant p = 0.042. C) Pyruvate decreased lipolysis when the L/P ratio was 10:1 p = 0.048, 5:1 p = 0.027 and pyruvate alone p = 0.015. P-values were calculated using ANOVA analysis with a post-hoc Dunnetts test.

In order to understand whether changes in the extracellular redox affected the turnover of triglycerides in fat cells we investigated their effect on breakdown of lipids through lipolysis as well as oxidation and esterification to complex lipids. We previously documented that scavenging ROS decreased lipolysis via an effect on translocation of HSL from the cytosol to lipid droplets, implicating ROS in the regulation of lipolysis [18]. In order to determine whether lipolysis was sensitive to external redox, glycerol release from human adipocytes was measured after 4 hours of incubation. Fig 3A shows that the addition of Acoc with or without βOHB, in ratios that achieved more oxidized conditions, increased glycerol release which reached significance at -337mV (p = 0.04) and Acoc alone (p = 0.042) when compared to control. These results were consistent with a causal relationship between an increase mitochondrial ROS production and stimulation of lipolysis.

Scavenging ROS with DPI [18] (Fig 3B) decreased lipolysis with control, Acoc, and βOHB by 25 ± 6.3%, 45 ± 3.3% (p = 0.0006), 16 ± 5.7% respectively and forskolin that was used as a positive control by 68 ± 2.7% (P<0.0001) (data not shown). Fig 3B shows that Acoc caused a significant increase (p = 0.001) in lipolysis as was shown in Fig 3A. We validated that DPI did not interfere in the assay by adding it to the standard curve and observing that there were no differences in fluorescence with or without the antioxidant (data not shown). In contrast, the addition of the more oxidized thiols (cystine, and GSSG) did not increase lipolysis (data not shown).

Pyruvate, like βOHB, significantly decreased lipolysis relative to lactate at -235 mV (p = 0.048), -224 mV (p = 0.027), and pyruvate alone (p = 0.015) (Fig 3C), consistent with its ability to enter mitochondria and diminish mitochondrial ROS production [25].

Forskolin induces lipolysis by increasing cAMP in adipocytes and was used as a positive control. As expected, forskolin increased lipolysis more than 3-fold. Average glycerol released by buffer was 285 ± 16.8 pmoles (n = 12) and by forskolin 975 ± 68.8 pmoles glycerol (n = 12). Due to the greater than 3-fold increase in lipolysis caused by forskolin, the forskolin-induced lipolysis data is not shown.

### Acetoacetate induced Lipogenesis

**Fig 4:**
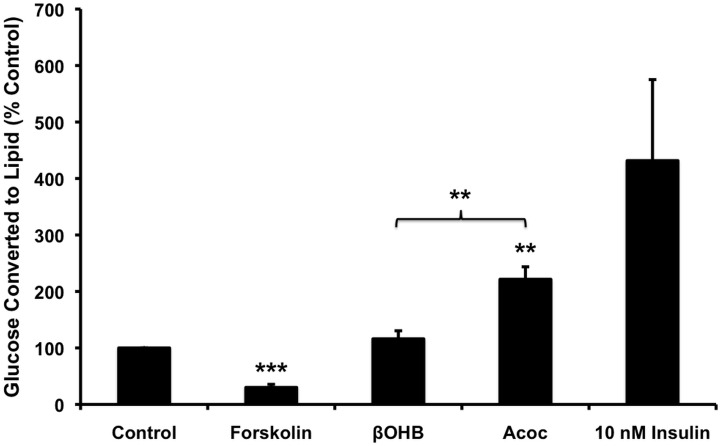
Acetoacetate induced Lipid synthesis. Fourteen days after differentiation, adipocytes were exposed to either 20 mM ßOHB and Acoc, 10 μM Forskolin, or 5 nM insulin (positive control) in a Krebs solution containing 5 mM glucose (of which 15 μM was ^14^C-glucose radiolabelled). After a 4 hour incubation period, cells were extracted in a chloroform-methanol. The organic layer (25 μl) was then placed in a LabLogic 300SL Liquid Scintillation counter (Brandon, Florida) to analyze β-particle emission to determine how much of the radiolabelled glucose was incorporated into lipid. Due to variations in the counts between experiments, data are represented as a percentage of the control condition average (41577±1630 counts). Data are presented as the mean ± SEM (N = 4). ANOVA analysis with a post-hoc Dunnetts test was used for statistics Forskolin p = 0.0001, Acoc p = 0.004.

It was recently reported that TG hydrolysis is required for β3-adrenergic receptor-induced *de novo* lipogenesis, TG turnover, and mitochondrial electron transport in all fat depots [26]. These findings suggest that lipolysis and synthesis are linked in adipocytes. We therefore used uniformly labeled [l-^14^C] glucose (0.67μmol/300 μCi/umol) as a substrate to measure the effects of βOHB/Acoc on lipogenesis. Fig 4 showed that addition of Acoc significantly (p = 0.004) increased glucose conversion to lipids compared with βOHB. The same results were found whether cells were incubated with radiolabeled glucose for 4 hours (Fig 4) or overnight (data not shown). Our data are in agreement with the ability of Acoc to form acetyl CoA, a precursor for lipogenesis [27–29]. Insulin (10 nM) was used as a positive control because it decreases lipolysis and increases malonyl-CoA formation, which is the precursor of fatty acid synthesis. As expected insulin increased and forskolin, which stimulates lipolysis, reduced lipogenesis.

### Ketone bodies did not affect fat oxidation

The effect of ketones on fatty acid oxidation and esterification was determined 4 hours after addition of ^14^C oleate (25 μM) bound to cyclodextrin. The distribution of labeled oleate into CO_2_ was taken as a measure of oxidation. Lipids were extracted from cells to determine fatty acid product distribution between the cell associated organic and the aqueous phases. Etomoxir, is an inhibitor of carnitine palmitoyltransferase 1 (CPT1) and fatty acid oxidation, and forskolin an inducer of lipolysis were used as negative and positive controls.

**Fig 5:**
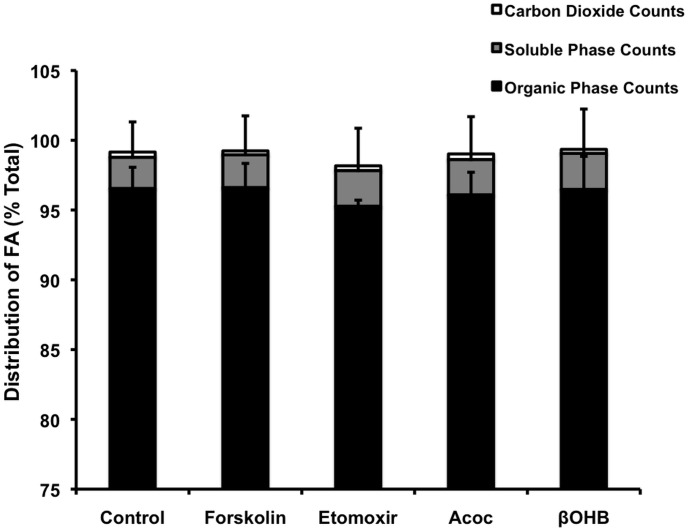
Ketone bodies did not affect fat oxidation or FFA distribution. Adipocytes were incubated for 4 hours in a Krebs buffer containing 15 μM ^14^C radiolabelled oleate in the presence of 20 mM ketone bodies, 30 μM etomoxir, 5 μM Forskolin or buffer control. Fat oxidation and FFA distribution were calculated as described under material and methods. For each sample, the data are presented as a percentage of the total counts (the sum of the counts from the organic layer, aqueous layer and filter paper) from 7 experiments done in triplicate. Label contained in each fraction was determined as described in methods.

Fig 5 shows that FA derived products accumulated in the cells but FA were oxidized very little and the conversion of exogenous oleate into CO_2_ was very small (0.2–0.6%). Addition of βOHB or Acoc had no effect on FA oxidation control 0.6% ± 0.08, βOHB 0.5% ± 0.1 and Acoc 0.5% ± 0.1. Low FA oxidation reflects the high capacity of adipose tissue to store triglycerides. These results indicated that the external redox state did not affect the rate of ß-oxidation. Consistent with our previous work, glucose is the main substrate responsible for ATP production in adipocytes [30].

As expected the major metabolic fate of FFA is esterification into TG and most of the counts were in the organic extract: control 99% ± 0.2, βOHB 98% ± 0.37 and Acoc 99% ± 0.2, indicating that the ketone bodies had no effect on this process or on the amount of FFA distributed into the soluble phase control 0.4% ± 0.02, βOHB 0.5% ± 0.04 and Acoc 0.4% ± 0.03 n = 6.

### βOHB increased mitochondrial O_2_ consumption through an increase in proton leak

**Fig 6:**
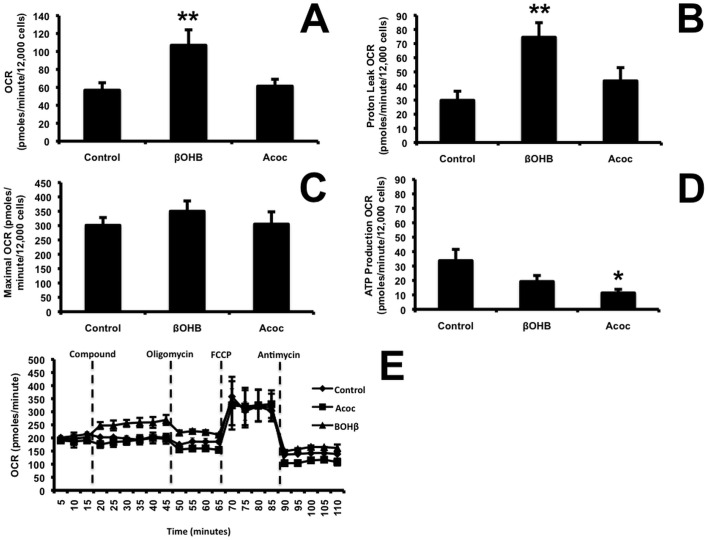
Addition of ßOHB, a more reduced ketone body increased maximal respiratory capacity and proton leak. Oxygen consumption rate was measured using the seahorse respiration assay for 20 min before and after addition of media (control), ßOHB or Acoc (20 mM) or respiratory inhibitors. A) For the first 20 min total cellular oxygen consumption (basal + non-mitochondrial respiration) was measured. Basal respiration is calculated from this quantity by subtracting non mitochondrial respiration. B) Oligomycin (10 μM) was added for 30 min to inhibit ATP synthase. The leak was calculated from the basal respiration which is not coupled to ATP synthesis. C) The maximal respiratory rate was determined by adding the uncoupler of oxidative phosphorylation, FCCP. D) ATP production was calculated from the decrease of oxygen consumption rate after the addition of Oligomycin A. E) Illustration of actual trace and sequence of additions used to calculate respiration. Data in A-D are represented as mean ± SEM (N = 7). ANOVA analysis with a post-hoc Dunnetts test was used for statistical analysis and showed that βOHB alone, significantly (p = 0.003) increased respiration and proton leak (p<0.044). ATP synthesis was significantly different (p = 0.04) between control and Acoc but not (p<0.09 between control and βOHB.

We previously showed in primary rat adipocytes, that ROS inhibits O_2_ consumption [31]. We therefore assessed the direct effect of βOHB, that decreased, and Acoc, that increased ROS, on mitochondrial respiration. O_2_ consumption rate (OCR) measurements were performed using the Seahorse analyzer. Fig 6A showed that βOHB alone, significantly (p = 0.003) enhanced respiration whereas Acoc did not, suggesting that ßOHB increased O_2_ consumption by increasing NADH or decreasing ROS to drive respiration. Oligomycin was used to inhibit ATP-linked respiration (oligomycin-sensitive fraction). The remaining component was due to a proton leak (oligomycin-insensitive fraction). As shown in Fig 6B, the proton leak was significantly (p = 0.044) increased only by βOHB. Following oligomycin addition, the maximal respiratory rate was determined by adding the uncoupler of oxidative phosphorylation, FCCP. Maximal respiration was similar among control, βOHB and Acoc (Fig 6C). This result ruled out the possibility that the increase in OCR induced by βOHB was due to increased fuel availability. The rate of O_2_ consumption from non-mitochondrial sources was determined by addition of antimycin A and was small and unchanged. The portion of respiration used to synthesize ATP was significantly lower (p = 0.04) with Acoc but did not achieve significance with βOHB (Fig 6D). Concentrations of inhibitors were selected by titration that produced optimal inhibition of respiration (data not shown).

Fig 6E shows a representative timecourse of oxygen consumption in response to exposure to injected compounds.

### Shifting redox state towards oxidative conditions early in adipogenesis in human adipocytes increased final lipid content

**Fig 7:**
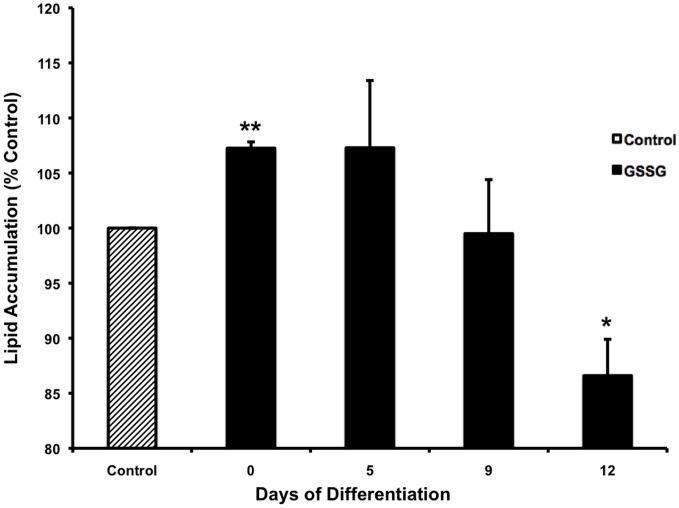
Shifting redox state towards oxidative conditions early in adipogenesis in human adipocytes increased final lipid content. Human preadipocytes were differentiated as detailed previously [17]. The media were supplemented with 55 μM GSSG. The induction media and the GSSG were added either on day 0, 5, 9 or 12 of differentiation. All cells were differentiated for a total of 14 days and fat accumulation was assessed on day 14 by Oil Red O staining by extracting the dye and quantifing as described [24]. Data were pooled from 2 experiments done with 8 replicates and expressed as % of control calculated as mean ± SD. The addition of GSSG at the start of differentiation significantly (p = 0.003) increased fat accumulation by day 14. On the other hand, addition of GSSG at day 12 significantly (p = 0.02) reduced lipid accumulation. ANOVA analysis with a post-hoc Dunnetts test was used for statistics.

There have been conflicting reports in the literature on the effect of ROS on adipogenesis [32,33]. We therefore tested whether redox changes induced by glutathione effected differentiation or final lipid accumulation. Fig 7 shows that the addition of GSSG, at the start of differentiation significantly (p = 0.003) increased fat accumulation by day 14. In contrast, supplementation of oxidized glutathione at day 5–9 of differentiation did not affect adipogenesis. On the other hand, addition of GSSG at day 12 significantly (p = 0.02) reduced lipid accumulation indicating that cells are effected differently at different stages of their life cycle by altered redox as suggested by Jones [34]. The effect of GSSG on fat accumulation was therefore, time- and differentiation-dependent. The addition of GSH did not have an effect on differentiation and the percent of control was 100.9 ± 2.5; 105.7 ± 4.2; 99.5 ± 4.5; 94.2 ± 6.1 when added at day 0, 5, 9 or 12 of differentiation.

## Discussion

### External redox modulates adipocyte function

Redox plays an important role in regulating crucial cellular events. Perturbations in redox correlate with pathological states such as cancer, cardiovascular disease, and metabolic disorders like obesity and T2D [1,35,36]. Oxidative stress is present in each of these conditions, as indicated by the presence of numerous oxidative biomarkers such as low reduced to oxidized thiol ratio or increased levels of peroxidation products (e.g. malondialdehyde). However, it is not clear whether this correlation is cause, effect or unrelated. Our previous studies on hepatocytes [16] and ß-cells [37], and the current studies on human adipocytes support the possibility that extracellular circulating redox changes can actually impact cellular function in an organ specific manner but through similar, ROS-related signals. Thus, a more oxidized state in liver impacts glucose production, in ß-cells increased ROS stimulate basal insulin secretion, and here we have shown that in adipocytes it impacted lipid turnover (Figs 3 and 4). Consistent findings were shown in hepatocytes [38], in cultured HUVEC [39], and in rat liver microsomes [40]. These results may be interpreted to indicate that oxidized redox potentials, due to ROS production occur as a consequence of limited capacity or depletion of the ROS scavenger pool.

### Pyruvate modulates both cytosolic and mitochondrial redox

**Fig 8:**
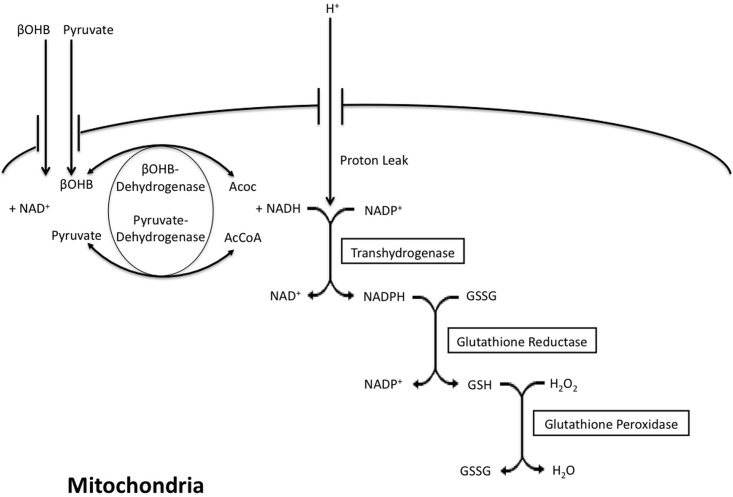
Model illustrating ROS and redox interactions in the mitochondria. Others have come to similar conclusions: the antioxidant activity of pyruvate was also shown by: O'Donnell-Tormey et al [41] who conclude that in mouse and human cells exogenous pyruvate, in concentrations that approximated physiological plasma and serum, protects cells from lysis by H_2_0_2_. Bassenge et al [42] also find in postischemic hearts, that pyruvate (0.1–5.0 mM) dose dependently inhibits ROS up to 80% while L-lactate (1.0–15.0 mM) stimulates both basal and postischemic ROS several fold. Thus cytotoxicities due to cardiac ischemia-reperfusion-generated ROS may also be alleviated by redox reactants such as pyruvate. Mallet et al [43] reported that pyruvate promoted robust contractile recovery of H_2_O_2_-challenged myocardium. Thus, studies with variation in the L/P ratio cannot help to differentiate cytosolic from mitochondrial targets. Further studies will require compartment specific alteration of ROS-scavenging enzymes to address this issue. In addition, we cannot differentiate between pyruvate directly scavenging ROS in a non-enzymatic manner [41] or through NADH or NADPH production. Regardless of the exact mechanism by which pyruvate inhibits lipolysis, it is clear that it involves reducing ROS generation (Fig 1D).

The initial goal in modulating the L/P ratio was to specifically control cytosolic redox and this was not achieved. The only redox pair that showed a discrepancy and reverse trend was the L/P in which the more oxidized pyruvate actually decreased ROS. However, whereas pyruvate can oxidize the cytosolic redox state, it increases NADH in the mitochondria. We interpret these data to indicate that the L/P couple cannot specifically influence only the cytosolic compartment and in fact we speculate that pyruvate may instead directly scavenge ROS either within or outside the mitochondria. Our previous work documented the ability of pyruvate to enter mitochondria, scavenge ROS and stimulate respiration [31]. An alternative possibility is that NADH generated by pyruvate in the mitochondria provides substrate for the transhydrogenase to produce the NADPH required for ROS scavenging by mitochondrial peroxidases. These speculative alternatives are illustrated in Fig 8.

### Ketones are both substrate and redox regulator

In these studies we used physiological redox carriers that also have functions other than defining redox status. Thus, the ßOHB/Acoc ratio reflects mainly hepatic mitochondrial redox state, however, ketones can also be important substrates in non-liver cells, including adipocytes, since they express both succinyl-CoA:3-ketoacid CoA transferase and acetoacetyl CoA synthetase [44]. Interestingly, ROS generation and several functional effects were similar to data obtained in hepatocytes where ketones cannot be used as substrate, suggesting that ROS levels could be the main determinant of observed effects under our conditions of nutrient sufficiency. Total ketone bodies were constant in our experiments with only the ratio being varied. Jain et al [39] using a cell free system found a dose dependent increase in superoxide anion generation by Acoc but not ß-hydroxybutyrate (SOD-inhibitable reduction of cytochrome C) and Acoc levels as low as 3.3 μmol/ml, similar to those frequently encountered in diabetic patients, can generate superoxide anion radicals. They also showed that elevated levels of Acoc constitute a risk factor for the oxidative modification of low-density lipoproteins and development of vascular disease in diabetic patients [45].

Thus, although we expect that ketones may make minor contributions as a fuel source, via succinyl-CoA:3-ketoacid CoA transferase, or complex lipid synthesis via acetoacetyl CoA synthetase, their main effect in these acute studies in the presence of glucose appears to be via their impact on redox and ROS.

### Compartmentation of redox couples

The rationale for using several different redox couples was based on the ability of changes in each couple to communicate such change to other couples (Fig 8 is an example) and on the compartmentation of several of redox functions. Since H_2_O_2_ equilibrates fairly rapidly, production in one compartment may be shared by others during the 1.5 to 4 h incubation period. Not all couples were tested on every function measured since our goal was to determine whether extracellular redox state could be communicated to intracellular compartments. Thus, our findings on ROS production were similar among the thiol and ketone redox couples. However, there were also differences: lipoperoxide production was not increased with Cys/CySS and lipolysis was only influenced by the ketones. These differences are intriguing and worthy of further investigation that may implicate localization of specific thiol reactions: mitochondrial regulation of lipolysis or specific glutathione-sensitive thiols in adipogenesis, although our data do not address these issues.

Cells have a variety of compartmentalized defense mechanisms to ameliorate the potential toxic effect derived from excessive ROS production. Superoxide dismutase (SOD) catalyzes the conversion of two superoxide anions into a molecule of H_2_O_2_ and oxygen. In the peroxisomes of eukaryotic cells, the enzyme catalase converts H_2_O_2_ to water and molecular oxygen. Glutathione peroxidases are a group of enzymes that also catalyze the degradation of hydrogen peroxide, as well as organic peroxides to alcohols and water. Glutathione is the most significant non-enzymatic oxidant defense mechanism. In humans it is found in relatively large amounts (mM) and serves to detoxify peroxides and regenerate a number of important antioxidants. Reduced glutathione (GSH) is regenerated from its oxidized form (GSSH) by the action of an NADPH dependent reductase. Interestingly while GSH is synthesized in the cell cytosol, its degradation occurs exclusively in the extracellular space and thus export from the cell is required for normal GSH turnover to ensure that both its intracellular and extracellular concentrations and the thiol-redox status are tightly regulated. After synthesis, some of the GSH is delivered into specific intracellular compartments including mitochondria and ER (reviewed in Ballatori 2009 [46] and Bachhawat AK 2013 [47] but most of it is extruded into the extracellular space.

Our results are in agreement with previously published data. Go and Jones [15] reported that in aortic endothelial cells derived from mice, a more oxidized extracellular redox potential (Eh) CySS elevates mitochondrial ROS production, activates nuclear factor NFkB, increases expression of adhesion molecules and stimulates monocytes binding to endothelial cells. Similar results were obtained by Imhoff et al [48]. NIH 3T3 cells were cultured in medium with extracellular Cys/CySS redox potentials (Eh), ranging from 0 to −150 mV. Cellular and mitochondrial ROS production significantly increased in cells incubated under more oxidizing extracellular conditions.

### ROS regulates lipid turnover

We previously documented that the mechanism by which ROS stimulates lipolysis involves altered translocation of hormone sensitive lipase from the cytosol to the lipid droplets and decreased lipolysis in the presence of the antioxidant DPI [18]. Here we showed that the addition of Acoc that induced both ROS and lipid peroxidation also increased lipolysis. In contrast, the addition of the more oxidized thiols (cystine, and GSSG) did not cause an increase in lipolysis above that of the reduced species. It has been reported in bovine subcutaneous adipocytes that ßOHB, but not Acoc inhibits both basal and noradrenaline stimulated lipolysis by inhibiting the formation of cyclic AMP [49]. Bjorntrop [50] reported that ßOHB inhibits the activation of hormone-sensitive lipase and monoglyceride lipase activities by norepinephrine in rat epididymal fat pad.

Our results showed that Acoc increased both lipolysis (Fig 3) and de novo lipogenesis (Fig 4) but not to the same extent. The increase in lipid synthesis far exceeded that of lipolysis. In addition our results showed that Acoc did not induce respiration above that of control (Fig 6).

Increased lipid synthesis by Acoc in subcutaneous human adipose tissue was reported by Kissebah [51]. They also found that at 20 mM, Acoc enhanced the incorporation of glucose into lipids.

Clearly in obesity, increased lipolysis must be balanced by increased lipid synthesis in order to sustain the excess fat mass. Our data are consistent with a model of increased lipid turnover under conditions of oxidative stress induced by variations in redox. However, our data cannot be interpreted quantitatively due to the fact that we did not separate the carbon in the glycerol moiety from that in the fatty acid or TG from phospholipid. The total counts included all 3, thus, a more precise determination of whether synthesis exceeded lipolysis would require a detailed separation of the different components.

### Regulation of the mitochondrial proton leak

Cortassa et al [52] used the term “Redox-Optimized ROS Balance” to describe how mitochondria play a role in energy provision while keeping ROS levels low enough to play a role in signaling. NADH donates electrons to both the respiratory chain and the antioxidant systems through transhydrogenation of NADH to NADPH (Fig 8). ßOHB conversion to Acoc by ßOHB dehydrogenase produces NADH and thus can have an effect on either ROS production via transhydrogenation, or respiration. Here we showed that respiratory flux was higher under reduced conditions compared with the more oxidized environment generated by Acoc suggesting that under reduced conditions the electrons donated by NADH are directed away from oxidative phosphorylation towards the antioxidant system to reduce ROS production and maintain levels that are compatible with physiological signaling.

Our results showed that the leak to maximum respiration ratio was significantly higher with ßOHB compared with control or Acoc. Because the proton leak was different after stimulation with ßOHB than Acoc it might indicate that the proton leak had an important function in the cell; potentially to prevent a dramatic increase in ROS. This interpretation is supported by the findings of Tieu et al [53] who showed that the infusion of βOHB in mice confers partial protection against dopaminergic neurodegeneration and motor deficits induced by mitochondrial permeability transition pore. Maalouf et al (2007) [54] reported that in rat neocortical neurons a combination of ßOHB and Acoc (1 mM each) significantly decreased mitochondrial production of ROS.

### Influence of ROS on adipogenesis

Conflicting data have been reported regarding the effect of ROS on adipogenesis. While ROS was found to increase levels of transcription factors and cell cycle progression and accelerate adipogenesis in 3T3-L1 adipocytes [32], pharmacological manipulation of mitochondrial ROS demonstrated negative correlation between ROS and differentiation in 3T3-F442A [33].

Similar to our findings, Imhoff et al [55] found that 3T3-L1 adipocytes produced more ROS under oxidized conditions, and that lipid accumulation and expression of early genetic markers of adipogenesis was enhanced under oxidizing conditions. In contrast Carriere [33] reported that differentiation of 3T3-F442A was inhibited by mitochondrial ROS-induced activation of the transcription factor CHOP-10/GADD153 which is a negative regulator of adipogenesis through interaction with C/EBPs.

We showed that adipogenesis, was indeed sensitive to the extracellular redox environment since the addition of GSSG, to early preadipocytes enhanced fat accumulation in mature adipocytes whereas the effect of later addition had no effect or was inhibitory.

In summary, our data support a powerful ability of extracellular redox to impact intracellular function in differentiated human adipocytes, although much additional work is ongoing to clarify the precise molecular species and compartments involved. Because of the correlation of redox changes with age and morbidity and the potential ability to modulate circulating redox with dietary intervention, this may be an important new target for disease prevention.

## Supporting Information

S1 Fig Comparison of lipolysis after 1 and 4 hour incubation time. Lipolysis was measured as glycerol release [18]. Adipocytes day 14 after differentiation were incubated in KRB with 0.5 mM oleate complexed to 150 μM BSA with or without the positive control Forskolin (5 μM) for either 1 or 4 hours. Aliquots were removed and the glycerol content was measured using an NADH-linked assay. Data were pooled from 3 experiments and repsesented as mean ± SE.
(TIF)
