# Supplementary figures and images for: Extracellular Redox Regulation of Intracellular Reactive Oxygen Generation, Mitochondrial Function and Lipid Turnover in Cultured Human Adipocytes
Source: PLoS One. 2016 Oct 14;11(10):e0164011. doi: 10.1371/journal.pone.0164011 (PMC5065187; doi:10.1371/journal.pone.0164011)

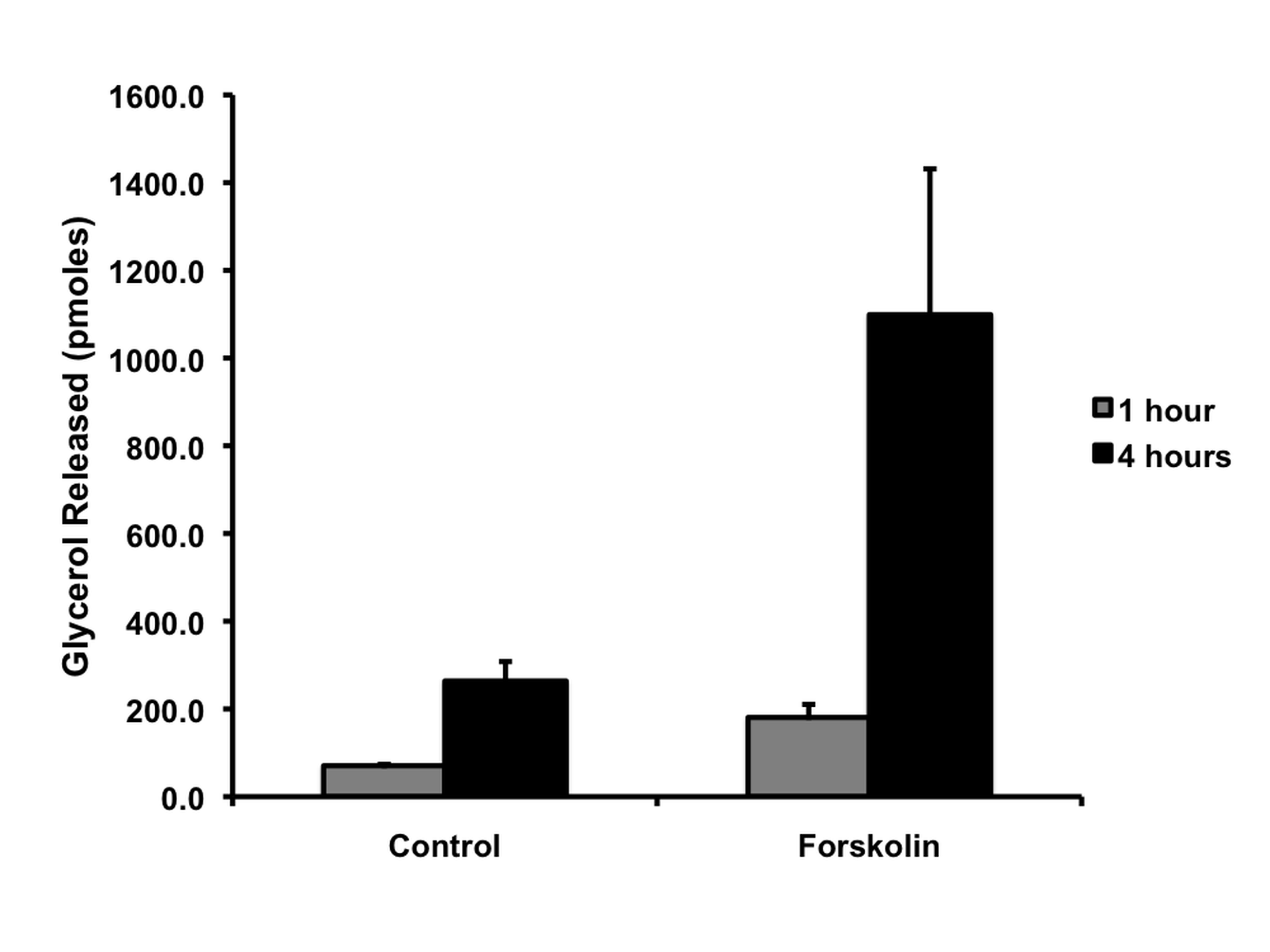

Supplement: S1 Fig — Lipolysis was measured as glycerol release [18]. Adipocytes day 14 after differentiation were incubated in KRB with 0.5 mM oleate complexed to 150 μM BSA with or without the positive control Forskolin (5 μM) for either 1 or 4 hours. Aliquots were removed and the glycerol content was measured using an NADH-linked assay. Data were pooled from 3 experiments and repsesented as mean ± SE. (TIF) [file pone.0164011.s001.tif]
